# Draft Genome Sequence of *Enterococcus faecalis* PC1.1, a Candidate Probiotic Strain Isolated from Human Feces

**DOI:** 10.1128/genomeA.00160-12

**Published:** 2013-02-21

**Authors:** Páraic Ó Cuív, Eline S. Klaassens, Wendy J. Smith, Stanislas Mondot, A. Scott Durkin, Derek M. Harkins, Les Foster, Jamison McCorrison, Manolito Torralba, Karen E. Nelson, Mark Morrison

**Affiliations:** a CSIRO Preventative Health Flagship Research Program, Queensland Biosciences Precinct, Queensland, Australia; b CSIRO Animal, Food, and Health Sciences, Queensland Biosciences Precinct, Queensland, Australia; c J. Craig Venter Institute, Rockville, Maryland, USA; d The Ohio State University, Columbus, Ohio, USA

## Abstract

*Enterococcus faecalis* is commonly isolated from the gastrointestinal tract of healthy infants and adults, where it contributes to host health and well-being. We describe here the draft genome sequence of *E. faecalis* PC1.1, a candidate probiotic strain isolated from human feces.

## GENOME ANNOUNCEMENT

*Enterococcus faecalis* is a facultative anaerobic bacterium that is a primary colonizer of the infant gastrointestinal tract and is also prevalent in the adult gastrointestinal tract (1–3). *E. faecalis* is typically considered to be a beneficial gut bacterium that has been shown to be capable of modulating the activities of specific animal pathogens (4, 5) and the host immune response (6, 7); consequently, several strains have been applied as probiotics for the maintenance of a healthy gut and the treatment of gut diseases, including diarrhea, irritable bowel syndrome, and ulcerative colitis (8–10). Despite this, *E. faecalis* is now also widely recognized as a potential gut pathogen that is capable of compromising the epithelial barrier (11), inducing inflammation in mouse models of experimental colitis (12, 13) and causing chromosomal instability in colonic epithelial cells (14, 15). Key intraspecies variations with relevance to pathogenesis exist, and thus, we sought to elucidate further the genomic potential of an *E. faecalis* isolate from a healthy human gut and to assess its potential as a probiotic candidate. We describe here the draft genome sequence of *E. faecalis* PC1.1, isolated as part of the Australian Human Gut Microbiome Project.

*E. faecalis* PC1.1 was isolated from a pooled fecal sample collected from healthy human subjects by plating aerobically on brain heart infusion (BHI) medium. Genomic DNA was prepared, and a 454 Life Sciences Genome Sequencer (GS) FLX system was used at the J. Craig Venter Institute (JCVI) to generate 2,753,924 bp of DNA sequence at 30× coverage. The individual sequence reads were assembled using the Newbler Assembler v2.3 to generate 79 contigs. The contig N_50_ was approximately 57.3 kb and the largest contig assembled was approximately 132.5 kb. Finally, the DNA sequences were annotated using the JCVI prokaryotic annotation pipeline.

The draft genome has a G+C content of 37.6% and contains 2,673 genes, with 2,619 protein-coding genes and 54 structural RNAs. As anticipated, the annotated genome of *E. faecalis* PC1.1 revealed the presence of factors relevant to the colonization of and persistence in the human gut, including protein orthologs with putative roles in mediating adhesion to host structural factors, including collagen, fibronectin, and fibrinogen. In addition, we identified a putative lectin with a suspected role in mediating adhesion to host structural glycans and also several proteins with likely roles in harvesting host glycans. The *E. faecalis* PC1.1 genome encodes a putative gelatinase and serine proteinase; however, it does not appear to encode an enterococcal surface protein or enterococcal cytolysin, which have been shown previously to be important for virulence.

This preliminary study provides insight into the genomic factors that contribute to the colonization of and persistence in the gut. Nontoxigenic *Clostridium difficile* can effectively colonize the gut of hamsters and humans and prevent colonization by toxigenic *C. difficile* strains (reviewed in reference 16); further studies will be necessary to determine the probiotic potential of *E. faecalis* PC1.1, including its ability to similarly prevent colonization and/or modulate the activities of pathogenic microbes and to productively influence the immune response.

### Nucleotide sequence accession numbers.

This Whole Genome Shotgun project has been deposited at DDBJ/EMBL/GenBank under the accession no. ADKN00000000. The version described here is the first version, ADKN01000000.
